# O.5.1-4 Approaches to promote active transport against the background of societal and climate change – Results of a nationwide survey from Germany

**DOI:** 10.1093/eurpub/ckad133.234

**Published:** 2023-09-11

**Authors:** Kristin Manz, Sarah Jane Böttger, Susanne Krug

**Affiliations:** Robert Koch Institute, Germany; manzk@rki.de; Robert Koch Institute, Germany; manzk@rki.de; Robert Koch Institute, Germany; manzk@rki.de

## Abstract

**Purpose:**

Active transport has various benefits, such as increasing physical activity levels and mitigating climate change. However, knowledge about common motives for active transport is limited, although it could be useful for designing effective public health strategies. In addition, the impact of currently observable increased remote working on active transport behaviour change are widely unknown. We aim to investigate (1) what motivates adults to walk or cycle to get to and from places, (2) differ health- and climate-related motives by sociodemographic factors (e.g., gender, age, education), and (3) whether an increase in remote working affects active transport behaviour.

**Methods:**

We used data from a nationwide population-based study conducted from 11/2021 till 02/2022. Our sample compromised 10,448 participants (51.1 % women), and data used were self-reported. We performed multivariable regression analyses to investigate whether sociodemographic factors predict the selection of a motive for active transport and whether a change in remote working predicts a change in active transport behaviour.

**Results:**

The most frequently chosen motives for active transport were “it is good for my health” (77.4 % of adults using active transport regularly) and “to be physically active” (75.7 %), followed by “it is good for the environment and climate” (54.8 %). The health-related motives were more often chosen by older adults, adults with a higher educational level and adults with a better health status. The motive to protect the environment and climate was more often chosen by women, adults with a higher educational level and adults with a better health status. A comparison between winter 2021/2022 and pre-pandemic times based on retrospective questions showed that an increase in remote working was associated with a reduction in active transport (relative risk ratio: 2.8) as well as an increase (relative risk ratio: 1.5; only significant in men).

**Conclusions:**

Health- and climate-related motives should be addressed when promoting active transport. Additionally, compensatory strategies are necessary to prevent a decline in total physical activity level due increased remote working.

**Support/Funding Source:**

The study was funded by the German Federal Ministry of Health.

